# A randomized, double-blinded, placebo-controlled clinical trial of sterile filtered human amniotic fluid for treatment of COVID-19

**DOI:** 10.1186/s12879-023-08856-y

**Published:** 2023-12-08

**Authors:** Joseph E. Tonna, Jan Pierce, Benjamin J. Brintz, Tyler Bardsley, Nathan Hatton, Giavonni Lewis, John D. Phillips, Chloe R. Skidmore, Craig H. Selzman

**Affiliations:** 1 Department of Surgery, Division of Cardiothoracic Surgery, University of Utah Health, Salt Lake City, UT USA; 2 Department of Emergency Medicine, University of Utah Health, Salt Lake City, UT USA; 3 Department of Medicine, Division of Hematology, Cell Therapy and Regenerative Medicine, University of Utah School of Medicine, Salt Lake City, UT USA; 4 Department of Medicine, Division of Epidemiology, University of Utah School of Medicine, Salt Lake City, UT USA; 5 Department of Medicine, Division of Epidemiology, University of Utah School of Medicine, Salt Lake City, UT USA; 6 Department of Medicine, Division of Pulmonary Medicine, University of Utah Health, Salt Lake City, UT USA; 7 Department of Surgery, Division of General Surgery, University of Utah Health, Salt Lake City, UT USA; 8 Department of Medicine, Division of Hematology, Cell Therapy and Regenerative Medicine, University of Utah School of Medicine, Salt Lake City, UT USA; 9 Department of Surgery, Division of Cardiothoracic Surgery, University of Utah Health, Salt Lake City, UT USA; 10 Department of Surgery, Division of Cardiothoracic Surgery, University of Utah Health, Salt Lake City, UT USA

**Keywords:** Human amniotic fluid, COVID-19, Coronavirus, SARS-CoV2, Inflammation

## Abstract

**Importance:**

Acellular human amniotic fluid (hAF) is an antimicrobial and anti-inflammatory fluid that has been used to treat various pro-inflammatory conditions. In a feasibility study, we have previously demonstrated that hAF could be safely administered to severely ill patients with coronavirus disease-19 (COVID-19). The impact of acellular hAF on markers of systemic inflammation and clinical outcomes during COVID-19 infection remain unknown.

**Objective:**

To determine the safety and efficacy of acellular, sterile processed intravenously administered hAF on markers of systemic inflammation during COVID-19.

**Design, settings and participants:**

This single-center Phase I/II randomized, placebo controlled clinical trial enrolled adult (age ≥ 18 years) patients hospitalized for respiratory symptoms of COVID-19, including hypoxemia, tachypnea or dyspnea. The study was powered for outcomes with an anticipated enrollment of 60 patients. From 09/28/2020 to 02/04/2022 we enrolled and randomized 47 (of an anticipated 60) patients hospitalized due to COVID-19. One patient withdrew consent after randomization but prior to treatment. Safety outcomes to 30 days were collected through hospital discharge and were complete by the end of screening on 6/30/2022.

**Interventions:**

Intravenous administration of 10 cc sterile processed acellular hAF once daily for up to 5 days vs placebo.

**Main outcome and measures:**

Blood biomarkers of inflammation, including C-Reactive protein (CRP), lactate dehydrogenase, D-dimer, and interleukin-6 (IL-6), as well as safety outcomes.

**Results:**

Patients who were randomized to hAF (*n* = 23) were no more likely to have improvements in CRP from baseline to Day 6 than patients who were randomized to placebo (*n* = 24) hAF: -5.9 [IQR -8.2, -0.6] vs placebo: -5.9 [-9.4, -2.05]; *p* = 0.6077). There were no significant differences in safety outcomes or adverse events. Secondary measures of inflammation including lactate dehydrogenase, D-dimer and IL-6 were not statistically different from baseline to day 6.

**Conclusions and relevance:**

In this randomized clinical trial involving hospitalized patients with COVID-19, the intravenous administration of 10 cc of hAF daily for 5 days did not result in statistically significant differences in either safety or markers of systemic inflammation compared to placebo, though we did not achieve our enrollment target of 60 patients.

**Trial registration:**

This trial was registered at ClinicalTrials.gov as #NCT04497389 on 04/08/2020.

**Supplementary Information:**

The online version contains supplementary material available at 10.1186/s12879-023-08856-y.

## Introduction

Typically associated with the highly morbid condition of amniotic fluid embolism during the peripartum period, amniotic fluid is a natural fluid with anti-inflammatory [1–4] and antimicrobial [5–7] properties. In lieu of discarding human amniotic fluid (hAF) after childbirth, it can be collected and processed to meet USP < 71 > sterility guidelines as a non-antigenic, anti-microbial, acellular fluid that has low demonstrated immunogenicity [8]. Purified hAF has been utilized for years under United States Food and Drug Administration (FDA) human cellular and tissue product (HCT/P) guidance 21 CFR 1271 [9] to treat a myriad of inflammatory conditions, including burns [10, 11], vascular and diabetic ulcers [12, 13], nerve regeneration [14]. In 2020, FDA regulations changed and purified hAF for this study was approved under Investigational New Drug #23,369 for use as an investigational therapeutic.

It was from these data and observations that we postulated intravenous administration of hAF might lead to reductions in serum markers of inflammation when administered to hospitalized patients with symptomatic Coronavirus Diseases 2019 (COVID-19), who were believed at the time to have a significant and possibly pathologic inflammatory response [15]. We first examined the safety and efficacy of hAF on inflammatory markers during COVID-19. Based on previous clinical use of hAF as an inhaled therapeutic for respiratory diseases, we undertook a 10 patients pilot feasibility study in COVID-19 patients [16]. Based on our findings, we undertook a larger Phase I/II study of purified hAF, administered intravenously, to treat the inflammatory effects of COVID-19 in patients requiring hospitalization [17].

## Materials and methods

### Trial design and oversight

The trial was a pragmatic, single center, blinded, placebo controlled, randomized clinical trial. The trial protocol has been previously published [17] and also appears in the Supplement. The trial was approved under University of Utah Institutional Review Board (IRB) Approval as #132,922 and was conducted under the principles of Declaration of Helsinki and in accordance with Good Clinical Practice Guidelines. The trial was financially supported, in part, by funding through the United States (US) CARES Act in response to the COVID-19 pandemic; the US Government had no role in the conduct or interpretation of the findings.

In accordance with IRB approval, patients were approached to obtain written informed consent prior to study enrollment or treatment. The trial was overseen by the Principal Investigator (PI). Adverse events were identified by study personnel, reviewed by the PI, then by a study monitor, who then made recommendations to a data and safety monitoring board (DSMB) who made final recommendations regarding study continuation or termination.

### Trial site and patients

The trial was conducted at the University of Utah Hospital from September 2020 until June 2022. Adults (age ≥ 18 years) admitted to the hospital were screened for enrollment. Briefly, patients were eligible if they were diagnosed as COVID-19 positive by a reverse transcriptase polymerase chain reaction (RT-PCR) test within 14 days of enrollment. These patients were symptomatic with COVID-19 (defined as cough, fevers, shortness of breath and/or sputum production), required supplemental oxygen *and* had a room air saturation of ≤ 94%, were not enrolled in other interventional trials, had a heart rate < 110 beats per minute and a blood pressure < 160/96 mm of mercury (mmHg), were able to consent and were willing to use accepted contraceptive methods for at least 90 days after administration of the last study drug dose.

As this was a Phase I/II trial in which we examined safety outcomes, and at the guidance of the FDA, we designed the trial to avoid enrolling patients who already had severe COVID-19 at enrollment, so as to minimize the chance that severe adverse events from COVID would confound the assessment of the intervention’s safety profile. Thus, patients were excluded if, prior to enrollment, they were already on mechanical ventilation (non-invasive or invasive), utilized chronic at home oxygen, were on home immunosuppressives, had impending respiratory failure (in the opinion of the investigator), had a hemoglobin < 9 g/deciliter (g/dL), chronic kidney disease (Stage ≥ 4), chronic heart failure (Class ≥ 3), durable left ventricular assist device, current thromboembolic phenomena, Type 2 or greater heart block, positive bacterial cultures, pericardial effusion or ascites, clinically significant arrythmias, liver function tests ≥ 3 × upper limit of normal, untreated human immunodeficiency virus, or other end-stage organ disease. Further details are given in the Supplement.

In February 2021, after the publication of the RECOVERY Trial, which showed a mortality benefit to dexamethasone during hospitalized COVID-19 [18], we removed a single exclusion criterion, which was any use of steroid immunosuppressives in the hospital prior to enrollment.

### Randomization

After signedconsent, the study team confirmed inclusion and exclusion criteria, and then enrolled the patient in the study. The study team would then notify unblinded study staff at Cell Therapy & Regenerative Medicine [CellReGen] (Salt Lake City, UT, USA) that the patient was enrolled. CellReGen study staff would confirm enrollment and immediately randomized the patient using the randomization module of REDCap (Nashville, TN, USA), which utilized a 1:1 allocation and permuted block randomization with blocks of size 2 and 4. Concealment was assured using this secure, centralized, web-based system. The study drug (hAF or saline placebo) was then prepared by CellReGen according to the randomization allocation, packaged as blinded study drug, and hand delivered to a study clinical research coordinator at the University of Utah Hospital. Drug preparation was done in accordance with US FDA IND regulation and has already been described [9]. Study team and PI remained blinded throughout enrollment until data collection closeout.

### Interventions

The intervention was 10 cc of intravenously administered hAF (or saline placebo), administered in blinded fashion, once daily for 5 consecutive days. Prior to clinical use, hAF undergoes a filtration, purification and sterilization process described in detail in the Supplement. The hAF utilized in this study has been previously described, but briefly, comes from one donor per dose (*i.e.*it is not pooled), and has been demonstrated to have a comparable balance and concentration of anti-inflammatory proteins across donors/doses [1]. The study drug was transferred to the clinical nurse for administration, with simultaneous direct oversight by the clinical research coordinator who obtained vital signs and monitored the patient for adverse events or reactions for 4 h after study drug administration, per the study protocol. All other clinical care was provided per standard clinical practice.

### Data collection and monitoring

Demographics and medical history were collected at baseline. Physical exam findings, vital signs, concomitant medications, and adverse events were collected daily from enrollment until day 6, then again at intensive care unit (ICU) discharge and at hospital discharge. Biomarker outcomes were collected at baseline and at day 6. Biomarkers were measured using the clinical laboratory at the University of Utah from blood drawn by clinical staff at the direction of study personnel. Patient records were utilized for follow up data as needed. Trial data were maintained on-site securely using paper records and REDCap. Any adverse events were reported by study personnel to the principal investigator. Severe adverse events were then reported to the medical monitor, and then to the DSMB within 3 days of occurrence.

Data were delivered securely at regular intervals to the blinded statistical analysis team, who prepared reports for DSMB review. At pre-specified intervals (after enrollment of 5, 20 and 40 patients), the DSMB convened to review study progress and outcomes and to recommended cessation or continuation of the study. DSMB also reviewed all severe adverse events and recommended cessation or continuation of the study.

### Outcomes

The primary outcome was the change in C-reactive protein (CRP) from prior to intervention until day 6, or last day of hospitalization. In cases of discharge from the hospital prior to day 6, if possible, the patient was brought back for biomarker assessment 6 days after first treatment.

Secondary outcomes included safety, adverse events and severe adverse events, death within 30 days, hospital length of stay, need for invasive mechanical ventilation, need for extracorporeal membrane oxygenation (ECMO), major adverse cardiac events, and change in inflammatory biomarkers, including interleukin-6, D-dimer, lactate dehydrogenase.

### Statistical analysis

Descriptive statistics were used to summarize the primary outcome, CRP, as well as secondary outcome measures by treatment group. Due to the primary outcome having a non-normal continuous distribution (as well as other continuous secondary outcomes), data was reported as medians and interquartile ranges for continuous variables rather than means and standard deviations. The primary analysis utilized the Wilcoxon-Mann–Whitney test to compare the difference scores of CRP (day 6 vs baseline measure) by treatment groups. Further analysis compared secondary outcome measures (lactate dehydrogenase U/L, D-dimer mg/mL, and interleukin-6 pg/mL) between treatment groups in a similar manner and utilized the Fisher’s Exact Test to compare the dichotomous safety outcome measures (death, intubation, ECMO, and major cardiac events) between treatment groups.

Descriptive statistics on patient demographic factors and hospital stay/admission characteristics were generated by treatment group as well as standardized difference scores (difference in means or proportions divided by standard error) to assess the balance between treatment groups at baseline.

### Sensitivity analyses

Two sensitivity analyses were performed. The first sensitivity analysis investigated the as-treated effect, by alternately defining the difference scores for continuous variables as the change from baseline to the last measure prior to discharge (rather than day 6 value). Similar statistics and tests were then carried out as described above. The second sensitivity analysis investigated a small imbalance in the baseline location of subjects (general hospital floor vs ICU). The analyses described above was repeated for the primary and secondary outcomes within the subgroup of subjects on the general hospital floor at baseline.

Two-sided tests with α = 0.05 were used to evaluated statistical significance. All analyses were performed in SAS Version 9.4.

### Sample size calculation

Assuming that patients in the hAF group completed the study without experiencing an AE, using the binomial distribution, we calculated that with a sample size of *n* = 30 patients per group (total *n* = 60), we would be able to state with 95% confidence that the safety of hAF is at least 90%. If there were 1, 2, or 3 subjects experiencing adverse events in the hAF group, we calculated that we would be able to state with 95% confidence that the safety of hAF is at least 85%, 80%, and 76%, respectively.

## Results

**Fig. 1:**
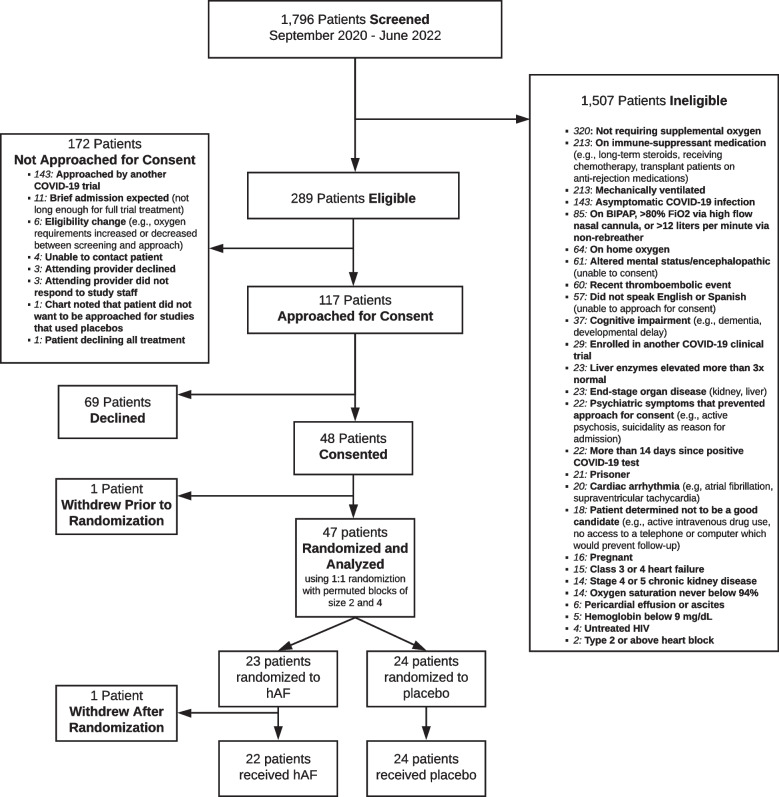
Study enrollment flowchart. Legend: Flowchart depicting the screening, enrollment, and randomization of patients

The trial screened 1,796 patients from 09/28/2020 until 06/30/2022. The trial was stopped early, prior to enrollment of 60 subjects, due to decreasing hospital admissions and increasing comorbidities (*e.g.* active cancer treatment, immune-suppressed transplant recipients) among admitted patients, which lead to ineligibility among admitted patients. Among screened patients, 172 met criteria for enrollment and 47 were enrolled and randomized from 10/28/20 until 02/2/22 (Fig. 1). One patient randomized to hAF withdrew consent after randomization, but prior to study drug administration, due to a dislike of phlebotomy, leaving 46 patients treated and for whom we collected clinical data. Analysis and outcomes are reported according to intention-to-treat (ITT) for all 47 randomized patients, regardless of receipt of study drug.

Patient clinical characteristics at baseline are listed in Table 1, split by intervention, with standardized difference scores. In our sample, standardized difference scores indicate small to medium potential imbalances between treatment groups in weight, race and hospital floor starting location. Patients randomized to receive hAF (*n* = 23) were more likely to be in the intensive care unit at enrollment (SDS 0.35), had measurable differences in race (SDS 0.57), and had lower median weight (SDS 0.24), than patients randomized to placebo (*n* = 24).

**Table 1:** Patient clinical characteristics at baseline

		**All Patients (*n* = 47)**	**hAF** **(*n* = 23)**	**Placebo (*n* = 24)**	**SDS**
**Age (years)**	Mean (Std)	57.8 (14.37)	56.8 (16.09)	58.8 (12.8)	0.13
**Sex**^**a**^	Male	21 (45)	11 (48)	10 (42)	0.12
	Female	26 (55)	12 (52)	14 (58)	
**Height (in)**	Median [IQR]	66 [65, 71]	66 [64, 72]	66 [65, 69]	-0.05
**Weight (lbs)**	Median [IQR]	212.6 [174.6, 244.7]	206 [174.6, 237.2]	216.9 [181.2, 253.8]	0.24
**Race**^**a**^	American Indian/ Alaskan Native	4 (9)	3 (13)	1 (4)	0.57
	Black	1 (2)	0 (0)	1 (4)	
	Hispanic	7 (15)	2 (9)	5 (22)	
	Multi-racial	3 (7)	2 (9)	1 (4)	
	White	31 (67)	16 (70)	15 (65)	
**Days from COVID-19 Onset to Study Day 1**	Median [IQR]	10 [8, 14]	11 [8, 15]	9 [8, 11.5]	-0.12
**Day 1 Location**^**a**^	Floor	38 (81)	17 (73.9)	21 (87.5)	0.35
	ICU	9 (19)	6 (26.1)	3 (12.5)	
**Patient admitted** ** to an ICU during hospitalization**^**a**^	No	34 (74)	14 (63.6)	20 (83.3)	-0.46
	Yes	12 (26)	8 (36.4)	4 (16.7)	
**Hospital LOS (days)**	Median [IQR]	5.5 [3, 8]	6 [4, 8.5]	5.5 [3, 8]	-0.05
**Study Day of ** **Discharge**^**a**^	2	4 (9)	3 (13.6)	1 (4.2)	-0.11
	3	7 (15)	1 (4.5)	1 (4.2)	
	4	7 (15)	4 (18.2)	3 (12.5)	
	5	3 (7)	1 (4.5)	2 (8.3)	
	6	25 (54)	13 (59.1)	12 (50)	
**Days Between CRP Measurements**	Median [IQR]	6 [6]	6 [6]	6 [5, 6]	-0.14
**Number of Treatment Doses Given**	Median [IQR]	5 [3, 5]	5 [3, 5]	5 [3, 5]	-0.06

*Abbreviations*: *SDS* Standardized difference score, *Std* Standard deviation, *in* Inches, *IQR* Interquartile range, *lbs* Pounds, *ICU* Intensive care unit, *LOS* Length of stay, *CRP* C-reactive protein ^a^n(%)

Patients were 58 (± 14) years old, 55% female, and 67% White. A median of 10 (IQR 8, 14) days had passed from symptom onset until Study Day 1. Nineteen percent of patients were located in the ICU at the time of enrollment. Median hospital length of stay was 5.5 (3, 8) days. Patients received a median of 5 (3, 5) doses of the study drug.

### Biomarkers

**Fig. 2:**
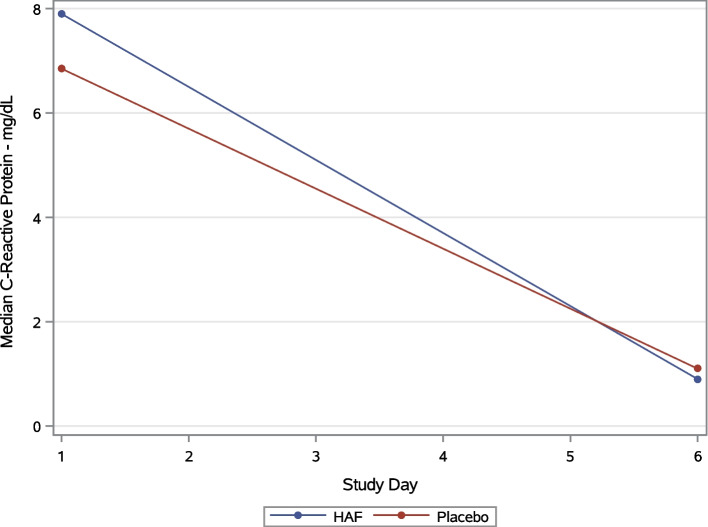
Plots of CRP values over study time. Legend: Median value of CRP (mg/dL) by group over study time. *Abbreviations:* CRP = C-reactive protein; mg = milligrams; dL = deciliter

**Fig. 3:**
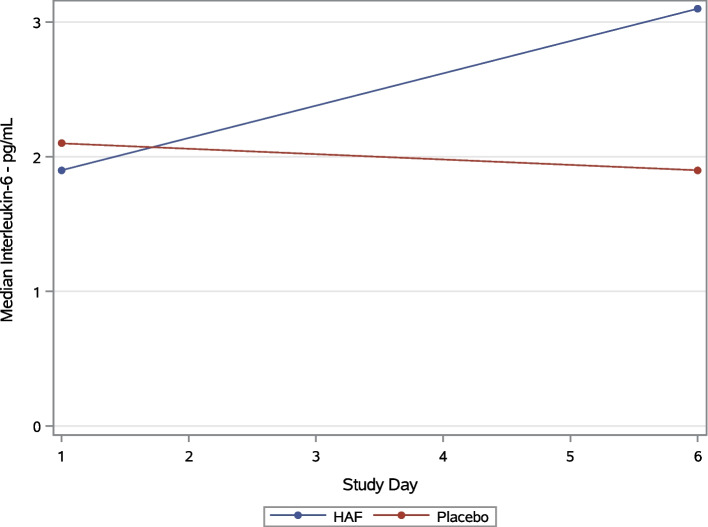
Plots of IL-6 values over study time. Legend: Median value of IL-6 (pg/mL) by group over study time. *Abbreviations:* IL-6 = interleukin-6; pg = picograms; mL = milliliter

Patients who were randomized to hAF were no more likely to have improvements in CRP from baseline to Day 6 than patients who were randomized to placebo (hAF: -5.9 (IQR -8.2, -0.6) vs placebo: -5.9 [-9.4, -2.05]; *p* = 0.61) (Fig. 2, Table 2). Likewise, changes in values of lactate dehydrogenase, D-dimer and IL-6 were not statistically different from baseline to day 6 (Fig. 3).

**Table 2:** Changes in CRP, LDH, D-Dimer, and IL-6 from baseline to day 6

Outcome	HAF Treatment Median (IQR)	Placebo Median (IQR)	*P*-Value
**Difference Between Day 6 and Baseline Value**			
C-Reactive Protein Change	-5.9 (-8.2, -0.6)	-5.9 (-9.4, -2.05)	0.6077
Lactate Dehydrogenase Change	-40.5 (-157, -20)	-57 (-95, -11)	0.8345
D-Dimer Change	0.1 (-0.1, 0.3)	-0.15 (-0.65, 0)	0.0586
Interleukin-6 Change	0 (0, 6.9)	0 (-1, 0)	0.0843
**Difference Between Day 6 and Baseline Value – Subset to Day 1 Floor Subjects**			
C-Reactive Protein Change	-5.9 (-8.2, -0.6)	-5 (-7.4, -1.8)	0.9591
Lactate Dehydrogenase Change	-58 (-151.5, -27.5)	-57 (-118.5, -26.5)	0.9310
D-Dimer Change	0.1 (-0.1, 0.3)	-0.1 (-0.4, 0)	0.0946
Interleukin-6 Change	0 (0, 3.6)	0 (-0.65, 0)	0.1424
**Difference Between Discharge (Day 6 or Early Discharge) and Baseline Value**			
C-Reactive Protein Change	-5.95 (-8.2, -1.3)	-5 (-9.2, -1.8)	0.7799
Lactate Dehydrogenase Change	-36 (-146, 16)	-42 (-75, 21)	0.4646
D-Dimer Change	0.1 (-0.1, 0.2)	-0.1 (-0.4, 0)	0.0397
Interleukin-6 Change	0 (0, 3.6)	0 (-0.85, 0)	0.0451
**Difference Between Discharge (Day 6 or Early Discharge) and Baseline Value – Subset to Day 1 Floor Subjects**			
C-Reactive Protein Change	-5.95 (-8.5, -0.95)	-4 (-7.4, -1.8)	0.8495
Lactate Dehydrogenase Change	-36 (-146, 16)	-42 (-75, -5)	0.5710
D-Dimer Change	0.1 (-0.15, 0.2)	-0.1 (-0.3, 0)	0.0720
Interleukin-6 Change	0 (0, 3.4)	0 (-0.6, 0)	0.0980

*Abbreviations*: *CRP* C-reactive protein, *LDH* Lactate dehydrogenase, *IL-6* Interleukin-6

### Safety and clinical outcomes

Among patients randomized to hAF, 2 patients (8.7%) died within 30 days, in contrast to 0 patients (0%) randomized to placebo (*p* = 0.23) (Table 3). Among patients randomized to hAF, 4 patients (17.4%) were intubated within 100 days, in contrast to 1 patient (4.2%) randomized to placebo (*p* = 0.19). One patient who received hAF had a major cardiac event (4.4%) vs 0 patients (0%) who received placebo (*p* = 0.49). No patients were placed on ECMO.

**Table 3:** Comparison of safety measures by randomized treatment group

Safety Outcome	hAF *n* (%)	Placebo *n* (%)	*P*-Value
Within 30 Days			
Death	2 (8.7)	0 (0)	0.2340
Within 100 Days			
Intubation	4 (17.4)	1 (4.2)	0.1882
ECMO	0 (0)	0 (0)	-
Major Cardiac Event	1 (4.4)	0 (0)	0.4894
**Subset to Day 1 Floor Subjects**			
Within 30 Days			
Death	1 (5.9)	0 (0)	0.4474
Within 100 Days			
Intubation	2 (11.8)	0 (0)	0.1935
ECMO	0 (0)	0 (0)	-
Major Cardiac Event	1 (5.9)	0 (0)	0.4474

When restricted to patients who were not in the ICU at enrollment, changes in CRP, LDH, D-dimer and IL-6 from baseline to Day 6 were not statistically different (Table 2).

Among patients randomized to hAF who were on the floor at enrollment, 1 patient (5.9%) died within 30 days, in contrast to 0 patients (0%) randomized to placebo (*p* = 0.4474). Among patients randomized to hAF, 2 patients (11.8%) died were intubated within 100 days, in contrast to 0 patients (0%) randomized to placebo (*p* = 0.19). One patient who received hAF had a major cardiac event (4.4%) vs 0 patients (0%) who received placebo (*p* = 0.4474). No patients were placed on ECMO.

When including last recorded values of biomarkers for patients who were discharged early and did not have a Day 6 value, d-dimer was more likely to have increased among patients randomized to hAF vs placebo (hAF: 0.1 (-0.1, 0.2) vs placebo: -0.1 (-0.4, 0); *p* = 0.04), as was IL-6 (hAF: 0 (0, 3.6) vs placebo: 0 (-0.85, 0); *p* = 0.045). Changes in CRP and LDH were not statistically different (Table 2).

When restricted to patients who were not in the ICU at enrollment and had last recorded values of their biomarkers used due to being discharged early from the hospital and not having Day 6 values obtained, changes in CRP, LDH, d-dimer and IL-6 from baseline to Day 6 were not statistically different (Table 2).

## Discussion

In this single center Phase I/II trial of patients requiring hospitalization due to COVID-19, daily intravenous administration of 10 cc of purified hAF for up to 5 days did not significantly decrease CRP compared to placebo, nor did it result in significant differences in major clinical outcomes, including death, intubation, major cardiac events, or ECMO utilization. Additionally, secondary outcomes of change in other blood biomarkers of inflammation, including LDH, D-dimer and IL-6 were not significantly different with administration of hAF.

A limitation to this study relates to the failure to achieve the pre-designated enrollment goal. At the end of the study period, the number of hospitalizations for patients with respiratory COVID-19 infections had dramatically decreased. Indeed, in the last 6 months of the study, only 2 patients were enrolled. In addition, during the nearly 2-year period of study, enrollment was challenged by many competing studies for this patient population at the University of Utah. Hence, with the guidance of the DSMB, it was decided to close the study and move forward with data analysis. To be clear, the study was halted not for safety reasons, but rather because of the logistical problem of study enrollment.

The ability to detect notable differences in the primary and secondary outcomes may not have been limited as much by under enrollment (47 of a planned 60) as by other factors, including a lack of effect of 10 mL of intravenous hAF on systemic markers of inflammation for hospitalized patients with COVID19. It was notable that in our study, the time from onset of symptoms until initiation of study drug was nearly 10 days. Quite possibly, the initial surge of inflammation associated with COVID-19 had already started to wane within a given patient, as it is known that inflammatory markers in patients with influenza achieve peak innate immune response by Day 5, with relative near resolution by Day 8 [19]. One could hypothesize that the administration of hAF was too late to impart differences in our predetermined outcome measures. Further, it was recently demonstrated that during COVID19, initial CRP levels determine distinct inflammatory profiles, which have distinct clinical courses and outcome of infection [20]. As we did not stratify or exclude based on initial CRP, we likely included disparate inflammatory profiles. Finally, and compared to our pilot trial, the majority of patients (81%) were bedded in acute care wards rather than the intensive care unit. Hence, it could also be hypothesized that the patients were not sick enough to detect appreciable difference in biomarkers or clinical outcomes, or that the dose tested was insufficient to elicit a biomarker response. Allowing the above considerations, the trial efficacy outcomes were non-significant and should be considered negative in the absence of other data.

Despite the lack of a significant difference in serum inflammatory biomarkers, the incidence of adverse events (AE) in all patients was lower than had been reported during hospitalization for COVID-19 early in the pandemic, which surprised us. This may have been reflective of the numerous exclusion criteria for comorbidities that the trial utilized, resulting in a less morbid population than was typically hospitalized for COVID. As described in the results, serious AEs (SAEs) in the trial that were observed included death, cardiogenic shock, hypotension and severe hypoxemia. There was no evidence from our analyses that these SAEs were more likely in the hAF group than in the placebo group; this conclusion was substantiated by the independent unblinded DSMB, which adjudicated each SAE after occurrence and recommended continuing the trial each time, finding the SAE attributable to the disease process and not the hAF therapy. Further, in planning our trial, we powered it based on the assumption that there would be no SAEs attributable to hAF. Our trial showed that 8 patients experienced SAEs (7 who received hAF and 1 who received placebo). While this difference was not statistically significant (*p* > 0.18 for SAE outcomes of death within 30 days; intubation, ECMO or major cardiac event within 100 days), we acknowledge it is higher. It should be noted though that, as mentioned, our rate of SAEs was lower than observed among patients hospitalized with severe COVID-19. Importantly, as our higher rate of SAEs occurring was higher than the 0 SAEs we had anticipated may have led to underpowering the trial. A large sample size may have indicated a significant difference in the primary outcome (CRP), other markers of inflammation, or in the safety outcomes. While the occurrence of AEs was numerically higher in the hAF group, the statistical bounds do not indicate that it was due to hAF, rather than chance.

To our knowledge, this study is the rigorous assessment of the effect of intravenous hAF on outcomes. Human AF has a long history of successful administration as an effective treatment for other inflammatory conditions or for augmenting regenerative healing, though it was administered intra- or transdermally in these other situations. To our knowledge, beyond our pilot, in which we administered hAF via inhaled and intravenous routes (cite), this is the first study to systematically assess the efficacy and safety of intravenous administration.

## Conclusions

In this single center Phase I/II randomized, double blinded, placebo-controlled trial, daily intravenous administration of 10 cc of sterile filtered, acellular human amniotic fluid for 5 days among patients hospitalized for COVID-19 did not result in statistically significant reductions in blood biomarkers of inflammation or efficacy outcomes. In addition, despite some of the stated limitation of the trial, this is the first study to demonstrate that intravenous hAF can be safely administered to hospitalized patients.

### Supplementary Information

**Additional file 1:** **Table S1.** Summary of outcome measures by randomized treatment group. **Table S2.** Summary of outcome measures by randomized treatment group (taking day 6 or early discharge value). **Table S3.** Summary of grade and relatedness of adverse events by randomized treatment group. **Table S4.** Sensitivity Analysis – Restricted to Subjects that start on the floor. **Figure S1.** Plots of LDH values over study time. Legend: Median value of LDH (U/L) by group over study time. *Abbreviations:* LDH =lactate dehydrogenase; U=Units; L=liter. **Figure S2.** Plots of d-dimer values over study time. Legend: Median value of d-dimer (mg/mL) by group over study time. *Abbreviations:* mg=milligrams; mL=milliliter.

## Data Availability

Available from the authors upon reasonable request. Please contact the corresponding author.

## Abbreviations

AE: Adverse event
COVID-19: Coronarvirus disease-19
CRP: C-reactive protein
DSMB: Data safety and monitoring board
ECMO: Extracorporeal membrane oxygenation
FDA: United States food and drug administration
hAF: Human amniotic fluid
ICU: Intensive care unit
IL-6: Interleukin-6
IRB: Institutional review board
LDH: Lactate dehydrogenase
mmHg: Millimeters of mercury
PI: Principal investigator
RT-PCR: Reverse transcription-polyamerase chain reaction
SAE: Serious adverse event
US CARES Act: United States Coronavirus aid, relief, and economic security act
USP: United States pharmacopeia
