# Supplementary material for: A randomized, double-blinded, placebo-controlled clinical trial of sterile filtered human amniotic fluid for treatment of COVID-19
Source: BMC Infect Dis. 2023 Dec 8;23:864. doi: 10.1186/s12879-023-08856-y (PMC10704623; doi:10.1186/s12879-023-08856-y)
Supplement: Supplementary file 1 — Additional file 1: Table S1. Summary of outcome measures by randomized treatment group. Table S2. Summary of outcome measures by randomized treatment group (taking day 6 or early discharge value). Table S3. Summary of grade and relatedness of adverse events by randomized treatment group. Table S4. Sensitivity Analysis – Restricted to Subjects that start on the floor. Figure S1. Plots of LDH values over study time. Legend: Median value of LDH (U/L) by group over study time. Abbreviations: LDH =lactate dehydrogenase; U=Units; L=liter. Figure S2. Plots of d-dimer values over study time. Legend: Median value of d-dimer (mg/mL) by group over study time. Abbreviations: mg=milligrams; mL=milliliter. [file 12879_2023_8856_MOESM1_ESM.docx]

**SUPPLEMENTAL ONLINE CONTENT**

Joseph E. Tonna MD MS, Jan Pierce MBA, Benjamin J Brintz, PhD, Tyler Bardsley, MS, Nathan Hatton, MD, Giavonni Lewis, MD, John D. Phillips, PhD, Chloe R. Skidmore, MS, Craig H. Selzman, MD. A Randomized, Double-blinded, Placebo-Controlled Clinical Trial of Sterile Filtered Human Amniotic Fluid for Treatment of COVID-19

Corresponding Author:

Craig H. Selzman, MD

craig.selzman@hsc.utah.edu

Division of Cardiothoracic Surgery

Department of Surgery

University of Utah Health

Salt Lake City, UT, USA

**Supplemental Methods (eMethods)**

1. eMethods
2. S Table 1: Summary of outcome measures by randomized treatment group
3. S Table 2: Summary of outcome measures by randomized treatment group (taking Day 6 or early discharge values)
4. S Table 3: Summary of grade and relatedness of adverse events by randomized treatment group
5. S Table 4: Sensitivity Analysis – Restricted to Subjects that start on the floor
6. S Figure 1: Plots of LDH values over study time
7. S Figure 2: Plots of d-dimer values of study time

**eMethods**

**Clinical processing of hAF prior to use**

CellReGen purifies and sterile filters amniotic fluid using a proprietary 40um to 0.2um serial filtration process which has been validated to provide sterility assurance levels for sterility under United States Pharmacopeia (USP) <71> guidance including batch sampling requirements, Bacteriostasis/Fungistasis validation and 14-day incubation reporting no growth. The blood of the donor (mother) is tested for infectious diseases, including HIV, Hepatitis B Virus, Hepatitis C Virus, Syphilis, West Nile Virus, Human T-lymphotropic viruses. The donor mother’s medical and social history are screened and the donor mother is determined to be eligible for birth tissue donation for transplant.

**S Table 1**

| Summary of outcome measures by randomized treatment group |
| --- |
|  |

|  | HAF (N = 23) - n (%) | | | | Placebo (N = 24) - n (%) | |  |
| --- | --- | --- | --- | --- | --- | --- | --- |
| Outcome Variables | Median (IQR) | |  | | Median (IQR) | |  |
| **C-Reactive Protein - mg/dL** | | | | | | |  |
| Pre-Treatment (Day 0) | 7.9 (4.8, 9.8) | |  | | 6.9 (2.5, 10.2) | |  |
| Post Treatment (Day 6) | 0.9 (0.4, 3.4) | |  | | 0.9 (0.3, 2.3) | |  |
| Difference (Post-Pre) | -5.9 (-8.2, -0.6) | |  | | -5.9 (-9.4, -2.1) | |  |
| **Interleukin-6 - pg/mL** | | | | | | |  |
| Pre-Treatment (Day 0) | 1.9 (1.9, 2.7) | |  | | 2.1 (1.9, 3.8) | |  |
| Post Treatment (Day 6) | 3.1 (1.9, 13.2) | |  | | 1.9 (1.9, 3.6) | |  |
| Difference (Post-Pre) | 0 (0, 6.9) | |  | | 0 (-1, 0) | |  |
| **D-Dimer - mg/mL** | | | | | | |  |
| Pre-Treatment (Day 0) | 0.8 (0.5, 1.4) | |  | | 0.8 (0.6, 1.4) | |  |
| Post Treatment (Day 6) | 1 (0.5, 1.9) | |  | | 0.8 (0.4, 1.2) | |  |
| Difference (Post-Pre) | 0.1 (-0.1, 0.3) | |  | | -0.2 (-0.7, 0) | |  |
| **Lactate Dehydrogenase - U/L** | | | | | | |  |
| Pre-Treatment (Day 0) | 421 (255, 505) | |  | | 370 (274, 472) | |  |
| Post Treatment (Day 6) | 315 (238, 391) | |  | | 285 (259, 365) | |  |
| Difference (Post-Pre) | -40.5 (-157, -20) | |  | | -57 (-95, -11) | |  |
| **Within 30 Days** | | | | | | |  |
| Death* | 2 (8.7) |  | | 0 (0) | |  | |
| **Up to 100 Days** | | | | | | |  |
| Intubation* | 4 (17.4) |  | | 1 (4.2) | |  | |
| ECMO* | 0 (0) |  | | 0 (0) | |  | |
| Major Adverse Cardiac Event* | 1 (4.3) |  | | 0 (0) | |  | |

*Mean (Standard Deviation)

| **S Table 2**  Summary of outcome measures by randomized treatment group (taking day 6 or early discharge value) |
| --- |

|  | HAF (N = 23) - n (%) | Placebo (N = 24) - n (%) |  |
| --- | --- | --- | --- |
| Outcome Variables | Median (IQR) | Median (IQR) | |
| **C-Reactive Protein - mg/dL** | | |  |
| Pre-Treatment (Day 0) | 7.9 (4.8, 9.8) | 6.9 (2.5, 10.2) | |
| Post Treatment (Day 6 or Early Discharge) | 1.1 (0.4, 6.6) | 0.8 (0.3, 2.2) | |
| Difference (Post-Pre) | -6 (-8.2, -1.3) | -5 (-9.2, -1.8) | |
| **Interleukin-6 - pg/mL** | | |  |
| Pre-Treatment (Day 0) | 1.9 (1.9, 2.7) | 2.1 (1.9, 3.8) | |
| Post Treatment (Day 6 or Early Discharge) | 1.9 (1.9, 9.9) | 1.9 (1.9, 2.9) | |
| Difference (Post-Pre) | 0 (0, 3.6) | 0 (-0.9, 0) | |
| **D-Dimer - mg/mL** | | |  |
| Pre-Treatment (Day 0) | 0.8 (0.5, 1.4) | 0.8 (0.6, 1.4) | |
| Post Treatment (Day 6 or Early Discharge) | 0.9 (0.4, 1.9) | 0.7 (0.4, 1) | |
| Difference (Post-Pre) | 0.1 (-0.1, 0.2) | -0.1 (-0.4, 0) | |
| **Lactate Dehydrogenase - U/L** | | |  |
| Pre-Treatment (Day 0) | 421 (255, 505) | 370 (274, 472) | |
| Post Treatment (Day 6 or Early Discharge) | 299 (238, 356) | 298.5 (265.5, 351) | |
| Difference (Post-Pre) | -36 (-146, 16) | -42 (-75, 21) | |

S Table 3

Summary of grade and relatedness of adverse events by randomized treatment group

|  |
| --- |
|  |
|  |

| Adverse Events |  | HAF (N = 23) n (column %) | Placebo (N = 24) n (column %) | P-Value |
| --- | --- | --- | --- | --- |
|  | | | |  |
| Grade | 1-2 (Mild/Moderate) | 13 (56.5) | 11 (45.8) | 0.4637* |
|  | 3-4 (Severe/Life-Threatening) | 3 (13) | 2 (8.3) | 0.6662 |
|  | 5 (Death) | 2 (8.7) | 0 (0) | 0.2340 |
|  | | | |  |
| Relatedness | Not Related | 4 (17.4) | 3 (12.5) | 0.7008 |
|  | Possibly Related | 12 (52.2) | 12 (50) | 0.8815* |
|  | Probably Related | 0 (0) | 0 (0) | NA |

| - Note: Percentage is out of total patients (in each treatment arm).  - P-value is obtained from the Fishers exact test unless marked with an * which denotes the Chi-Squared Test |
| --- |

S Table 4

Sensitivity Analysis – Restricted to Subjects that start on the floor

| Adverse Events |  | HAF (N = 17) n (column %) | Placebo (N = 21) n (column %) | P-Value |
| --- | --- | --- | --- | --- |
|  | | | |  |
| Grade | 1-2 (Mild/Moderate) | 7 (41.2) | 10 (47.6) | 0.6913* |
|  | 3-4 (Severe/Life-Threatening) | 2 (11.8) | 2 (9.5) | 1.0000 |
|  | 5 (Death) | 1 (5.9) | 0 (0) | 0.4474 |
|  | | | |  |
| Relatedness | Not Related | 1 (5.9) | 2 (9.5) | 1.0000 |
|  | Possibly Related | 7 (41.2) | 11 (52.4) | 0.4916* |
|  | Probably Related | 0 (0) | 0 (0) | NA |

- Note: Percentage is out of total patients (in each treatment arm).

- P-value is obtained from the Fishers exact test unless marked with an * which denotes the Chi-Squared Test

**S Figure 1**

**Title:** Plots of LDH values over study time

**Legend:** Median value of LDH (U/L) by group over study time

*Abbreviations:* LDH =lactate dehydrogenase; U=Units; L=liter

**S Figure 2**

**Title:** Plots of d-dimer values over study time

**Legend:** Median value of d-dimer (mg/mL) by group over study time

*Abbreviations:* mg=milligrams; mL=milliliter
